# Microcephaly in north-east Brazil: a retrospective study on neonates born between 2012 and 2015

**DOI:** 10.2471/BLT.16.170639

**Published:** 2016-11-01

**Authors:** Juliana Sousa Soares de Araújo, Cláudio Teixeira Regis, Renata Grigório Silva Gomes, Thiago Ribeiro Tavares, Cícera Rocha dos Santos, Patrícia Melo Assunção, Renata Valéria Nóbrega, Diana de Fátima Alves Pinto, Bruno Vinícius Dantas Bezerra, Sandra da Silva Mattos

**Affiliations:** a The Heart Circle-Royal Portuguese Hospital, Av. Agamenon Magalhães 2760, Paissandu, Recife, Pernambuco, CEP 52010-902, Brazil.

## Abstract

**Objective:**

To assess the number of children born with microcephaly in the State of Paraíba, north-east Brazil.

**Methods:**

We contacted 21 maternity centres belonging to a paediatric cardiology network, with access to information regarding more than 100 000 neonates born between 1 January 2012 and 31 December 2015. For 10% of these neonates, nurses were requested to retrieve head circumference measurements data from delivery-room books. We used three separate criteria to classify whether a neonate had microcephaly: (i) the Brazilian Ministry of Health proposed criterion: term neonates (gestational age ≥ 37 weeks) with a head circumference of less than 32 cm; (ii) Fenton curves: neonates with a head circumference of less than −3 standard deviation for age and gender; or (iii) the proportionality criterion: neonates with a head circumference of less than ((height/2))+10) ± 2.

**Findings:**

Between 1 and 31 December 2015, nurses obtained data for 16 208 neonates. Depending on which criterion we used, the number of neonates with microcephaly varied from 678 to 1272 (4.2–8.2%). Two per cent (316) of the neonates fulfilled all three criteria. We observed temporal fluctuations of microcephaly prevalence from late 2012.

**Conclusion:**

The numbers of microcephaly reported here are much higher than the 6.4 per 10 000 live births reported by the Brazilian live birth information system. The results raise questions about the notification system, the appropriateness of the diagnostic criteria and future implications for the affected children and their families. More studies are needed to understand the epidemiology and the implications for the Brazilian health system.

## Résumé

**Objectif:**

Estimer le nombre d'enfants nés avec une microcéphalie dans l'État de Paraíba, au nord-est du Brésil.

**Méthodes:**

Nous avons contacté 21 maternités qui faisaient partie d'un réseau de cardiologie pédiatrique et qui avaient accès à des informations concernant plus de 100 000 enfants nés entre le 1 er janvier 2012 et le 31 décembre 2015. Pour 10% de ces enfants, nous avons demandé aux infirmières de récupérer les données sur les mesures du périmètre crânien dans les registres des salles d'accouchement. Nous avons utilisé trois critères distincts pour déterminer si des nouveau-nés présentaient une microcéphalie: (i) le critère proposé par le ministère de la Santé brésilien: enfants nés à terme (âge gestationnel ≥ 37 semaines) avec un périmètre crânien inférieur à 32 cm; (ii) les courbes de Fenton: nouveau-nés au périmètre crânien inférieur à un écart type de −3 pour leur âge et leur sexe; ou (iii) le critère de proportionnalité: nouveau-nés au périmètre crânien inférieur à ((taille/2)+10) ± 2.

**Résultats:**

Entre le 1er et le 31 décembre 2015, les infirmières ont recueilli des données sur 16 208 nouveau-nés. En fonction du critère utilisé, le nombre de nouveau-nés présentant une microcéphalie variait de 678 à 1272 (4,2–8,2%). Deux pour cent (316) des nouveau-nés remplissaient tous les critères. Nous avons observé des fluctuations temporelles de la prévalence de la microcéphalie à partir de la fin de l'année 2012.

**Conclusion:**

Le nombre de cas de microcéphalie indiqué ici est bien plus élevé que celui de 6,4 pour 10 000 naissances vivantes déclaré par le système d'information brésilien sur les naissances vivantes. Ces résultats soulèvent des questions quant au système de notification, à la pertinence des critères de diagnostic et aux implications futures pour les enfants concernés et leur famille. Il est nécessaire de réaliser d'autres études afin d'en comprendre l'épidémiologie et les implications pour le système de santé brésilien.

## Resumen

**Objetivo:**

Evaluar el número de niños nacidos con microcefalia en el estado de Paraíba, en el noreste de Brasil.

**Métodos:**

Se contactó con 21 centros de maternidad pertenecientes a una red de cardiología pediátrica con acceso a información relativa a más de 100 000 neonatos nacidos entre el 1 de enero de 2012 y el 31 de diciembre de 2015. Para el 10% de estos neonatos, se solicitó a las enfermeras que recuperaran los datos acerca de las mediciones del perímetro cefálico de los archivos de las salas de parto. Se utilizaron tres criterios distintos para clasificar si un neonato tenía o no microcefalia: (i) los criterios propuestos por el Ministerio de Sanidad de Brasil: recién nacidos a término (edad gestacional ³ 37 semanas) con un perímetro cefálico inferior a 32 cm.; (ii) las curvas de Fenton: recién nacidos con perímetro cefálico inferior a la desviación estándar de -3 para la edad y el género; o (iii) los criterios de proporcionalidad: recién nacidos con un perímetro cefálico inferior a ((altura/2)+10) ± 2.

**Resultados:**

Entre el 1 y el 31 de diciembre de 2015, las enfermeras obtuvieron datos relativos a 16 208 neonatos. Según los criterios que se utilizaron, el número de recién nacidos con microcefalia varió de 678 a 1 272 (4,2–8,2%). El 2% (316) de los neonatos cumplió con los tres criterios. Se observaron fluctuaciones temporales de prevalencia de microcefalia a partir de finales de 2012.

**Conclusión:**

Las cifras registradas de casos de microcefalia son mucho mayores del 6,4 por cada 10 000 nacidos vivos registrados por el sistema de información de nacidos vivos de Brasil. Los resultados plantean dudas acerca del sistema de notificación, la aptitud de los criterios de diagnóstico y las implicaciones futuras para los niños afectados y sus familias. Es necesario realizar más estudios para comprender la epidemiología y las implicaciones para el sistema sanitario brasileño.

## ملخص

**الغرض:**

تقييم عدد الأطفال الذين ولدوا وهم يعانون من مرض صغر الرأس في ولاية باريبا بشمال شرق البرازيل.

**الطريقة:**

قمنا بالتواصل مع 21 من مراكز رعاية الحوامل قبيل الولادة التابعة لشبكة علاج أمراض القلب لدى الأطفال، إضافة إلى الحصول على معلومات متعلقة بعدد يزيد عن 100,000 من حديثي الولادة في الفترة ما بين 1 يناير 2012 و 31 ديسمبر 2015. وقد طُلب من طواقم التمريض الحصول على البيانات الخاصة بقياسات دوران الرأس من سجلات غرف الولادة بالنسبة لـ 10% من حديثي الولادة هؤلاء. استخدمنا ثلاثة معايير منفصلة لتصنيف ما إذا كان حديث الولادة يعاني من مرض صغر الرأس: (أ) المعيار الذي اقترحته وزارة الصحة البرازيلية: حديثي الولادة بتمام الحمل (العمر الحملي ≥ 37 أسبوعًا) الذين يقل مقاس دوران الرأس لديهم عن 32 سم؛ أو (ب) منحنيات فنتون: حديثي الولادة الذين يقل مقاس دوران الرأس لديهم عن -3 بحسب الانحراف المعياري للعمر والنوع؛ أو (جـ) معيار التناسب: حديثي الولادة الذين يقل مقاس دوران الرأس لديهم عن ((الارتفاع/2)+10) ± 2.

**النتائج:**

حصلت طواقم التمريض على بيانات تخص 16,208 من حديثي الولادة في الفترة ما بين 1 و 31 ديسمبر/كانون الأول 2015. واختلف عدد حديثي الولادة المصابين بمرض صغر الرأس من 678 إلى 1272 (بنسبة 4.2 إلى ‏8.2%) استنادًا إلى كل معيار من المعايير التي استخدمت. توفرت جميع المعايير الثلاثة في نسبة تمثل اثنين بالمئة من حديثي الولادة (316). لقد قمنا بملاحظة التقلبات الزمنية لمدى انتشار مرض صغر الرأس من أواخر العام 2012.

**الاستنتاج:**

إن أعداد المواليد المصابين بمرض صغر الرأس والذين تم الإبلاغ عن حالاتهم هنا أعلى من 6.4 لكل 10,000 من المواليد على قيد الحياة الذين تم الإبلاغ عنهم بواسطة نظام المعلومات البرازيلي الخاص بالمواليد على قيد الحياة. وتساهم النتائج في إثارة التساؤلات حول نظام الإخطار عن حالات الإصابة، ومدى ملاءمة معايير التشخيص، والتأثيرات المستقبلية المترتبة على الأطفال المصابين وعائلاتهم. هناك حاجة إلى إجراء المزيد من الدراسات لفهم المرض الوبائي والتأثيرات المترتبة على النظام الصحي البرازيلي.

## 摘要

**目的:**

评估巴西东北部帕拉伊巴州先天小头症儿童的数目。

**方法:**

我们联系了 21 个隶属儿科心脏病学网络的妇产中心，访问了 100 000 多名出生于 2012 年 1 月 1 日至 2015 年 12 月 31 日之间的新生儿的信息。经要求，护士从产房图书中检索了 10% 新生儿的头围数据。 我们采用三个不同的标准来界定新生儿是否患有小头症： (i) 巴西卫生部提出的标准: 足月新生儿（胎龄 37 周）头围小于 32 厘米；(ii) 芬顿曲线： 新生儿头围与同龄同性别婴儿头围相比，标准偏差小于 3；或 (iii) 比例标准： 新生儿头围小于 【（身高/2）+ 10】 ± 2。

**结果:**

2015 年 12 月 1 日至 31 日期间，护士收集了 16 208 名新生儿的数据。 根据我们使用的标准，患有小头症的新生儿的数量从 678 增加至 1272 名（4.2——8.2%）。 百分之二 （316） 的新生儿完全符合这三个标准。 我们观察到从 2012 年年底开始，小头症患病率有短暂性波动。

**结论:**

此处报道的小头症患儿的数量要远远高于巴西出生信息系统所报道的每 10 000 名新生儿中有 6.4 名患儿的数量。 此结果引发了人们对通知系统、诊断标准的适宜性以及对患病儿童及其家庭的未来影响的质疑。 还需要进一步开展研究以了解流行病学及其对巴西卫生系统的影响。

## **Цель** Оценить количество детей, рожденных с микроцефалией, в штате Парайба на северо-востоке Бразилии.

**Методы:**

Авторы обратились в 21 родильный дом, относящийся к сети детских кардиологических больниц, и получили доступ к информации о более чем 100 000 случаев новорожденных в период между 1 января 2012 года и 31 декабря 2015 года. У 10% этих новорожденных авторы попросили медицинских сестер проверить сведения журналов учета родильных залов об окружности головы. Использовались три отдельных критерия микроцефалии: (i) критерий, предложенный Министерством здравоохранения Бразилии: доношенный новорожденный (срок беременности ≥ 37 недель) с окружностью головы менее 32 см; (ii) кривые Фентона: новорожденные с окружностью головы, которая на −3 стандартных отклонения меньше средних значений для своего возраста и пола; (iii) критерий пропорциональности: новорожденные, чья окружность головы меньше значения, полученного по формуле ((рост/2)+10) ± 2.

**Результаты:**

В период с 1 по 31 декабря 2015 года медицинские сестры получили данные для 16 208 новорожденных. В зависимости от использованных критериев количество новорожденных с микроцефалией варьировало от 678 до 1272 (4,2–8,2%). Два процента (316) новорожденных отвечали всем трем критериям. Начиная с конца 2012 года наблюдались временные флуктуации частоты микроцефалии.

**Выводы:**

Количество случаев микроцефалии в этом регионе значительно выше, чем цифры, приводимые в системе информации о рождении живых детей в Бразилии (6,4 на 10 000 живых новорожденных). Эти результаты поднимают вопросы о системе уведомления, о пригодности диагностических критериев и о том, какие последствия ожидают таких детей и их родителей в будущем. Необходимы новые исследования для понимания этой эпидемиологии и ее последствий для системы здравоохранения Бразилии.

## Introduction

Congenital microcephaly is a neurological condition defined by an occipital-frontal head circumference that is smaller than expected for the gestational age and gender. Head circumference is a validated measurement for intracranial brain volume, since the growth of the cranium depends on the forces of the expanding brain. Microcephaly could, therefore, be used as an indicator of an undersized brain. However, controversy exists concerning the suitable lower limit for this measurement, as well as over the need for ethnically controlled data.^1^ Furthermore, establishing the clinical implications of an undersized brain is difficult. Common causes of microcephaly are genetic disorders, severe malnutrition during pregnancy and intrauterine infections – such as syphilis and toxoplasmosis.

Microcephaly is a rare condition. In the United States of America, the prevalence has been estimated to range from 2.0 to 12.0 newborns with microcephaly per 10 000 live births^2^ and the European Surveillance of Congenital Anomalies centre reports 2.9 newborns with microcephaly per 10 000 live births.^3^ In Brazil, the live birth information system, SINASC, reported a prevalence of 0.6 newborns with microcephaly per 10 000 live births in 2010.^4^ However, the reporting of microcephaly was neither compulsory nor had clearly defined criteria. Between November 2015 and February 2016, however, the reported number of newborns with microcephaly reached a total of 5280 in 25 of the 27 Brazilian states. More than 80% of them were from north-east Brazil and the State of Paraíba reported 776 newborns with microcephaly.^5^

Since 2012, the government in Paraíba has run a paediatric cardiology network,^6^ in collaboration with *Círculo do Coração*, a nongovernmental organization. The network has screened and stored cardiovascular data from more than 100 000 neonates from this state. However, the original data set does not include head circumference data. To increase the knowledge about microcephaly prevalence in Paraíba, the network carried out a four-week exercise between 1 and 31 December 2015, to obtain head circumference data from 10% of the neonates in the data set. This article summarizes the results from this research.

## Methods

The network collected data on all neonates born in the 21 participating maternity centres. To retrieve data on head circumference for a subset of these neonates, we exported data – including an identification number, the date of birth and the mother’s name, for all neonates in the network – from the database to an electronic spreadsheet. We added an extra column to the spreadsheet with the heading head circumference. We sent the spreadsheet to each participating centre by email, and in the email asked the nurses to retrieve the head circumference measurements from the delivery-room books. In Brazil, a newborn’s occipital-frontal head circumference is measured in the delivery room by a nurse or a paediatrician, who uses a common measuring tape. The measurement is noted in the delivery room book as part of the routine perinatal practice in Paraíba state.

The goal was to retrieve data on approximately 10% of the neonates born between 1 January 2012 and 31 December 2015. To distribute the collection of data evenly over time, we suggested that the nurses should randomly select 10% of each month’s deliveries. The nurses first printed the spreadsheet and wrote down the head circumference of the selected neonates on the list. Then, they logged on to the network’s database, typed in the identification number for each selected neonate and manually entered the measurement in the newly added field for the head circumference data.

We obtained additional information about the mother’s name and address, the gestational age, the length and weight, and the gender of the neonate at birth, from the network’s database. Data on ethnicity were not collected. We only included neonates with complete data.

We classified neonates as having microcephaly by using one of the following criteria: (i) the criterion proposed by the Brazilian Ministry of Health, for which a term neonate (gestational age ≥ 37 weeks) is diagnosed with microcephaly if the head circumference is less than 32 cm;^7^ (ii) Fenton curves, for which neonates are classified as having microcephaly if the head circumference is less than −3 standard deviation for age and gender;^4^ or (iii) the proportionality criterion, for which a neonate is classified as having microcephaly if the head circumference is less than ((height/2)+10) ± 2.^8^

We made four different analyses: one for each criterion and one where the neonates had to fulfil all three criteria. In addition, within each of the criterion, a neonate was classified as having severe microcephaly when the measurement was −3 standard deviation or more for that criterion.

We used the software R, version 3.3.0 (R Foundation, Vienna, Austria) for all data analysis. For statistical analysis we used the Friedman test.

## Results

Nurses retrieved data from 16 208 neonates, of whom 7750 were females and 8458 were males. Most of the babies (15 591; 96.2%) were full term neonates, 12 146 (74.9%) weighed more than 3000 g and 15 572 (96.1%) measured more than 45 cm in length, at birth (Table 1).

**Table 1:** Characteristics of the neonates born between 1 January 2012 and 31 December 2015, Paraíba, Brazil

Characteristic	No (%) (*n* = 16 208)
**Gender**	
Female	7 750 (47.8)
Male	8 458 (52.2)
**Gestational age, weeks**	
< 32	21 (0.1)
≥ 32– < 34	39 (0.2)
≥ 34– < 37	557 (3.4)
≥ 37	15 591 (96.2)
**Weight, g**	
< 1500	37 (0.2)
≥ 1500– < 2000	107 (0.7)
≥ 2000– < 2500	731 (4.5)
≥ 2500– < 3000	3 187 (19.7)
≥ 3000	12 146 (74.9)
**Length, cm**	
< 35	9 (0.1)
≥ 35– < 40	58 (0.4)
≥ 40– < 45	569 (3.5)
≥ 45– < 50	9 415 (58.1)
≥ 50	6 157 (38.0)
**OFC, cm**	
≤ 30	229 (1.4)
> 30– ≤ 31	376 (2.3)
> 31– ≤ 32	958 (5.9)
> 32– ≤ 33	2 185 (13.5)
> 33	12 460 (76.9)

OFC: occipital-frontal head circumference.

Notes: Nurses retrieved data on head circumference for 10% of neonates participating in the paediatric cardiology network in Paraíba. Inconsistencies arise in some values due to rounding.

Depending on which criterion we used, 4.2% (678) to 8.2% (1272) of neonates were classified as having microcephaly and 316 (2.0%) neonates fulfilled all three criteria. Of the neonates that fulfilled all three criteria, three were classified as having severe microcephaly (Table 2).

**Table 2:** Occurrence of microcephaly in neonates born between 1 January 2012 and 31 December 2015, Paraíba, Brazil

Criteria	No. (%) (*n* = 16 208)
**Brazilian Ministry of Health^a^**	
Normal	14 319 (91.4)
Microcephaly	
All	1 272 (8.2)
Severe	16 (0.1)
**Fenton curves^b^**	
Normal	15 530 (95.8)
Microcephaly	
All	678 (4.2)
Severe	6 (< 0.1)
**Proportionality criterion^c^**	
Normal	15 405 (95.0)
Microcephaly	
All	803 (5.0)
Severe	11 (< 0.1)
**Fulfilling all criteria**	
Normal	15 876 (98.0)
Microcephaly	
All	316 (2.0)
Severe	3 (< 0.1)

^a^ The proposed criterion from the Brazilian Ministry of Health is that a term neonate (gestational age 37 weeks or more) is diagnosed with microcephaly if the head circumference is less than 32 cm. Of the neonates in the data set, 15 591 were term neonates.

^b^ Fenton curves classify neonates as having microcephaly if head circumference is less than −3 standard deviation for age and gender.

^c^ The proportionality criterion classifies neonates as having microcephaly if the head circumference is less than ((height/2)+10) ± 2.

Note: Inconsistencies arise in some values due to rounding.

**Fig. 1:**
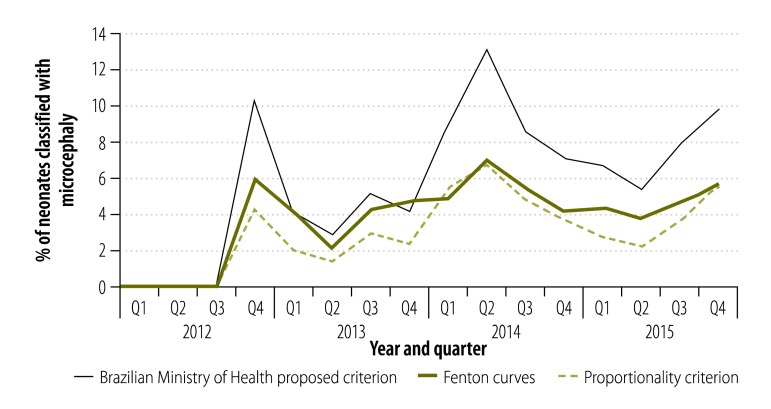
Temporal distribution of neonates with microcephaly in Paraíba, Brazil, 2012–2015

Q1: first quarter; Q2: second quarter; Q3; third quarter; Q4: fourth quarter.

Notes: The proposed criterion from the Brazilian Ministry of Health is that a term neonate (gestational age 37 weeks or more) is diagnosed with microcephaly if the head circumference is less than 32 cm. Fenton curves classify neonates as having microcephaly if head circumference is less than −3 standard deviation for age and gender. The proportionality criterion classifies neonates as having microcephaly if the head circumference is less than ((height/2)+10) ± 2.

**Fig. 2:**
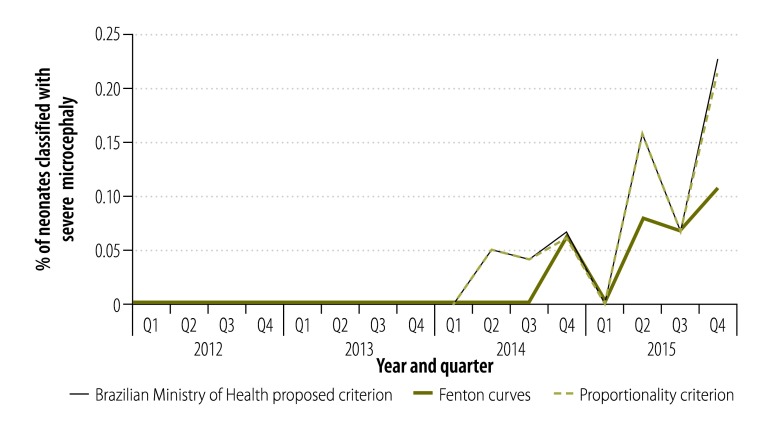
Temporal distribution of neonates with severe microcephaly in Paraíba, Brazil, 2012–2015

Q1: first quarter; Q2: second quarter; Q3; third quarter; Q4: fourth quarter.

Notes: The proposed criterion from the Brazilian Ministry of Health is that a term neonate (gestational age 37 weeks or more) is diagnosed with microcephaly if the head circumference is less than 32 cm. Fenton curves classify neonates as having microcephaly if head circumference is less than −3 standard deviation for age and gender. The proportionality criterion classifies neonates as having microcephaly if the head circumference is less than ((height/2)+10) ± 2.

Between 2012 and 2015, the number of neonates with microcephaly fluctuated, all of the criteria showed a similar pattern over this time. From the end of 2012, the numbers were higher than expected, with the highest peak in mid-2014 (Fig. 1). When we only considered neonates with severe microcephaly, we observed a significant increase in numbers (*P* = 0.001) from the third quarter of 2015 (Fig. 2).

## Discussion

Between 2012 and 2015, the Brazilian live birth information system reported a microcephaly prevalence of 6.4 per 10 000 live births in Paraíba.^9^ Our study indicates that the prevalence could have been even higher. When projecting our findings to the total number of live births in Paraíba in 2014 (*n* = 58 147), if we use the proposed criterion from the health ministry, the estimated number of neonates born with microcephaly in that year is 4652; if we use the Fenton curves, the estimated number is 2442; and if we use the proportionality criterion, the estimated number is 2907. The estimated number of neonates fulfilling all three criteria is 1105.

These observations raise several questions. First, what is the true prevalence of microcephaly in north-east Brazil? The discrepancy in numbers between the Brazilian Ministry of Health and this study may reflect underreporting in recent years associated with an even greater incidence of microcephaly than presumed. It is possible that a high prevalence of the non-severe forms of microcephaly had been occurring before the current outbreak, but health workers had only notified the live birth information system about the cases of neonates with typical severe phenotypes. As a result of the number of neonates with severe microcephaly increasing in the last quarter of 2015, the health workers' awareness might have increased, therefore causing them to notify the live birth information system about the milder forms as well.

Second, what is the most appropriate diagnostic criterion for microcephaly in this setting? If we only consider the number of neonates with severe microcephaly in our study, this number is in the expected range of 2–12 neonates with microcephaly per 10 000 live births, reported by the United States Centers for Disease Control and Prevention.^2^ However, most of the neonates in our study had milder forms of microcephaly. The clinical significance of these milder forms is not well established. For example, we do not know if a head circumference of 31 m or 32 cm in a term neonate could be within normal limits for this particular population. We also do not know if ethnical or nutritional factors could explain these findings. We need to determine if the population is facing large numbers of children with a neurological disease or if the observation is an anthropometric variation of normality.

Third, what is the cause of the increase in microcephaly prevalence? This question has attracted a lot of attention and the most likely explanation is the Zika virus outbreak that started in mid-2014 in Brazil. It is believed that the Zika virus was introduced to Brazil during the 2014 FIFA World Cup.^10^ Researchers have postulated a possible association between microcephaly and the Zika virus intrauterine infection.^11^ Evidence favouring this hypothesis are: perinatal transmission of the Zika virus;^12^ the virus’s strong neurotropism;^13^ and the detection of the virus in amniotic fluid of fetuses with microcephaly.^11^

The Zika virus was identified in Africa more than 50 years ago^14^ and despite the numerous outbreaks of the virus, both inside and outside Africa, an increase in the number of neonates with microcephaly has not been reported. However, during a Zika virus outbreak in French Polynesia in 2013, the virus was associated with several conditions, including Guillain Barré syndrome and microcephaly.^12^^,^^15^

The Zika virus is transmitted by *Aedes aegypti* mosquitoes and infections transmitted by this vector demonstrate temporal fluctuations similar to the fluctuations of microcephaly that we present here.^16^ Researchers are considering whether other concurrent infections transmitted by *Ae. aegypti*, such as dengue and chikungunya, might explain the increase in microcephaly seen in Brazil. The hypothesis is that concurrent infections have an additive effect that promotes microcephaly. A study from the island of La Réunion– an overseas department of France – showed an association between chikungunya infections and microcephaly.^17^ Teratogens’ exposure – such as vaccines or drugs used in early pregnancy – is another potential factor to consider as a potential cause of microcephaly.^1^^,^^18^ Furthermore, malnutrition, which has previously been associated with microcephaly, could worsen the effect of other etiological factors. Indeed, most of the reported neonates with microcephaly have come from low-income families.^6^

In Brazil, controlling the *Ae. aegypti* vector has been a major public health strategy to combat the arboviruses. This strategy is justified both by its potential to reduce the number of babies born with microcephaly – if the association with Zika virus infections proves true – and for the reduction of other *Ae. aegypti* transmitted diseases, such as dengue and chikungunya infections.

At this stage, we can only conclude that Brazil is facing a new and challenging public health problem. The current epidemiological and clinical data are insufficient to make conclusions concerning the risk factors of microcephaly and the pathogenic mechanisms of the Zika virus. Further retrospective studies and follow-up investigations on children with well-defined or borderline microcephaly will be important to clarify the etiology as well as the neurological consequences of these diagnoses. Children born with microcephaly can also have other birth defects, which could further aggravate the neurological manifestations. For the affected children and the families it is paramount that the government provides management strategies, such as social inclusion programmes and access to specialized health care.
